# Thermal Plasma Synthesis of Different Alloys and Intermetallics from Ball Milled Al-Mo and Al-Ni Powder Systems

**DOI:** 10.3390/ma15238646

**Published:** 2022-12-04

**Authors:** Khashayar Khanlari, Inès Esma Achouri, Francois Gitzhofer

**Affiliations:** Département de Génie Chimique et de Génie Biotechnologique, Université de Sherbrooke, 2500 Boulevard de l’Université, Sherbrooke, QC J1K 2R1, Canada

**Keywords:** plasma system, Al-Mo system, Al-Ni system, powder, intermetallics, material synthesis

## Abstract

Lightweight alloys have great importance for car manufacturers that aim to produce safer, lighter, and more environmentally friendly vehicles. As a result, it is essential to develop new lightweight alloys, with superior properties to conventional ones, respecting the demands of the market. Al and its alloys are good candidates for reducing the overall weight of vehicles. The objective of this research was to understand the possibility to synthesize different Al alloys and intermetallics by implementing the plasma system and using two different Al-Ni and Al-Mo powder systems. This was done by separately injecting non-reacted raw Al-Ni and Al-Mo composite powder systems into the plasma reactor. In the first step, the milling parameters were optimized to generate Al-Ni and Al-Mo composite powders, with sizes over about 30 µm, having, respectively, a homogeneous mixture of elemental Al and Ni, and Al and Mo in their particles. Each of the composite powders was then injected separately into the plasma system to provide conditions for the reaction of their elements together. The obtained Al-Ni and Al-Mo powders were then studied using different methods such as scanning electron microscopy, X-ray diffractometry, and energy dispersive X-ray analysis. Regardless of the initially used powder system, the obtained powders were consisting of large spherical particles surrounded by a cloud of fine porous particles. Different phases such as Al, AlNi_3_, Al_3_Ni_2_, and AlNi were detected in the particles of the Al-Ni powder system and Al, Mo, AlMo_3_, MoO_3_, and MoO_2_ in the Al-Mo powder system.

## 1. Introduction

Lightweight alloys are and will be of great importance in the next decade for car manufacturers [1]. Cars are expected to be safer, pollute less, and be adapted to electric propulsion with batteries and fuel cells. Electric cars have new types of engines or power generators under their hood that make them heavier than regular gasoline engines [2]. Car manufacturers must then reduce the weight of the other parts of these cars to preserve their performance. However, the safety of vehicles should not be reduced or sacrificed. This is a major reason for developing new materials having superior properties as compared to conventional ones.

The use of Al-based alloys is important for reducing the overall vehicle weight. This is because these alloys are about 40% lighter than steel. This contributes to fuel efficiency, fewer and cleaner emissions, and improved performances. Moreover, as compared to steel, Al-based alloys can absorb two times more energy and therefore they offer very good protection to passengers. This means that it is possible to produce a lighter vehicle without sacrificing its safety. Other than its light weight and strength, Al is recyclable, corrosion resistant, durable, ductile, and shows good formability and conductivity. Considering these, it is important to develop different new Al-based materials and explore their properties [1,3,4].

In this research, the possibility of the combinatorial synthesis of different phases and materials in two different binary systems of Al-Ni and Al-Mo was investigated. Based on the equilibrium phase diagrams of these binary systems, many different intermetallics and phases could be generated between the elements existing in each of these systems. Al, Al_3_Mo, AlMo_3_, Al_8_Mo_3_, and Al_4_Mo are a few examples of phases that could be produced in the Al-Mo system, and Al, AlNi_3_, Al_3_Ni_2_, Al_3_Ni, and AlNi are examples of those that could be generated in the Al-Ni system [5,6]. Each of these phases might have promising properties and be potentially useful for different applications. The other reason for choosing Al-Mo and Al-Ni systems was that the differences between the melting points of Mo and Ni and that of Al are significant; additionally, even the melting point of Mo is almost similar to the boiling point of Al (2470 °C). The melting points of Mo, Ni, and Al are 2623 °C, 1455 °C, and 660.3 °C, respectively. This allows us to develop an unconventional alloy synthesis method for metals exhibiting deep discrepancies in their physical properties.

One approach to studying each of these above-mentioned phases is to target to produce that specific phase separately by different processing routes, and then test and characterize it. However, this conventional approach is quite slow and time-consuming. The approach taken in this research was to apply a processing route, making us able to generate and obtain different phases at the same time and in a combinatorial mode. This will save a lot of time in the process of studying different phases and understanding their properties [7,8].

The first application of the combinatorial approach to the discovery of new solid-state materials with novel physical or chemical properties was reported by Xiang et al. [9]. Xiang et al. [9] generated libraries by sputtering target materials through physical masks, using an RF magnetron sputtering gun. Combinatorial synthesis or screening is a way of automatically synthesizing libraries of hundreds or even thousands of tiny samples of gradually changing composition, each to be tested for some property. A paper by Xiang et al. [10] focusing on high-temperature superconductors stimulated some rapid innovations in the field and led to the conduction of subsequent works. Xiang et al. [10] have since then produced a definitive overview of what they call combinatorial materials synthesis and screening of functional materials. Writing in Advanced Engineering Materials, Zhao [11] described how he has applied the principles of the combinatorial approach to the assessment of structural load-bearing materials such as superalloys used in the hot parts of jet engines. In addition, this paper described a rapid method of determining phase diagrams. These are just a few examples showing the advantages of this combinatorial method and since the mid-1990s, the same approach has been applied to the search for new materials with desirable properties including catalytic, luminescent, superconducting, magnetoresistant, or ferroelectric properties [12,13]. Many recent studies have focused on processing new phases and materials from elementally mixed powders using different techniques. Parts having multiphase structures were obtained in these studies [14,15,16,17,18].

In this research, this approach was implemented by applying an in-flight thermal plasma technology to simultaneously synthesize different light Al-based metallic materials. In the first step of this research, a complete investigation was conducted using a ball mill apparatus to agglomerate the elemental powder particles and obtain both Al-Ni and Al-Mo composite powders as starting materials. This part of the project consisted of the optimization of milling time for the Al-Ni and Al-Mo systems. These included: following the milling procedure, the particles must have been larger than about 30 µm and have a good homogeneity of the Al and Ni components inside each composite grain of Al-Ni and Al and Mo inside each composite grain of Al-Mo powder. In addition, as we aim to investigate the possibility to produce materials and reactions between the elements during the plasma process, it is required that the milling process did not cause any reaction between the different elements existing in the powder systems. The ratios between the Al and Ni or Al and Mo were 1. Following the milling optimization, the plasma spraying of the prepared dry powders was carried out using a radio frequency inductively coupled plasma (RF-ICP) system. High operating temperature of the plasma (over 10^4^ K), high specific enthalpy (up to 10 MJ/ kg, depending on which central gas is being used), a wide area of the relatively high-temperature region (~30–40 mm in diameter), and a low plasma flow velocity (~20–50 m/s) are some of the advantages of the RF-ICP process that make it a desirable technique with the potential to lead in the reaction between the inserted elements [19,20]. The synthesized products were then characterized separately using scanning electron microscopy (SEM), X-ray diffractometry (XRD), energy dispersive X-Ray analysis (EDX), and elemental mapping.

To the best of our knowledge, no systematic research has yet been done on this topic and the approach taken in this project is quite novel. Success in this project will provide the know-how to simultaneously develop and obtain different novel materials using the thermal plasma system and from the elementally mixed powders.

## 2. Materials, Machines Used, and Characterization Methods

### 2.1. Characterization of the Initial Powders

The commercially elemental Al (99.80% Al content), Mo (99.95% Mo content), and Ni (99.90% Ni content) powders were purchased respectively from Alfa Aesar, Haverhill, MA, USA, Atlantic Equipment Engineers, Upper Saddle River, NJ, USA, and Novamet, Lebanon, TN, USA. These powders were used to prepare the Al-Ni and Al-Mo starting powder systems. The morphology of the elemental powders (based on the emissions received from the backscattered electrons (BSE)) in their original purchased conditions was characterized by a JEOL-JSM-840A scanning electron microscopy (SEM) machine equipped with an electron dispersive X-ray analysis (EDX) (Tokyo, Japan). The average particle sizes of the powders were determined using a laser diffraction particle sizing measurement machine (Malvern Mastersizer 3000, Malvern, UK). The O and C impurity contents of the powders were determined by inert gas fusion analytical instrument Leco machines. Note that these tests, impurity content and average particle size measurements, were done on three different powder batches and the average results are reported in this manuscript.

### 2.2. Mechanical Agglomeration of the Powders and Characteristics of the Plasma Spraying Machine

To uniformly mix the two-component elemental particles in the Al-Mo and Al-Ni powder systems before feeding them to the plasma reactor, a mechanical agglomeration apparatus was used. This apparatus consisted of a metal jar that could be rotated in a three-dimensional random movement. The jar cavity was filled with mixed elemental powders to be treated and two metal balls. The ratios between the Al and Ni or Al and Mo in each of these powder systems were 1. The volume of the jar was about 50 mL, the total volume of the balls was 12.5 mL (25% of the total volume of the jar) and the powder volume was 1.25 mL (10% of the volume of the balls). An addition of 0.5 mL of methanol was also used to avoid powder agglomeration on the milling balls. Different milling times of 6 min, 1 h, 3 h, 5 h, and 10 h were used to agglomerate the powders. These milling processes were each conducted several times to determine the reproducibility of the results.

During the rotation movement of the jar, the heavy balls induce shock phenomena on the powder particles and the powder is thought to be either trapped between the two balls or trapped between a ball and the wall of the jar [21]. The optimum milling time causing the generation of agglomerated Al-Mo and Al-Ni powders with sizes larger than about 30 µm and good homogeneity of the two-component elemental particles was determined in this research.

The particle size distributions of the particles were analyzed by a software called SigmaScan Pro. SigmaScan Pro is an advanced image analysis application that allows you to capture, modify, enhance, and measure digital images. In this research, the images were taken using an optical microscope and then analyzed by this software.

The only information asked from the software was the diameter of the particles. Each of the powder sets (Al-Mo and Al-Ni) is expected to contain a number of large particles and a number of very small particles. As a result, the standard deviation of the resulting data is expected to be high. To avoid a large standard deviation, these two particle types (in each powder set) were analyzed separately by the software and the results are reported here.

The experimental dry-injection plasma system, employed for the plasma spraying program, consisted of three major components: an induction plasma torch installation, a powder synthesis reactor, consisting of two large cylinders, and a powder feeding system. Operation of the overall system is managed from the control panel; this includes switches for initiating the gas discharge and the circulated cooling water flows, the controls for modulation of plate power, chamber pressure and adjustment of the flow rates of the various gases employed, as well as the controls for all the auxiliary components’ power supplies. Note that Ar and/or He gases were employed in this research as the central, sheath, and carrier gases.

The induction plasma torch (Tekna Plasma System Inc., Sherbrooke, QC, Canada) is designed to employ a water-cooled, ceramic plasma confinement tube, in which a 4-turn induction coil, connected through a tank circuit to the radio frequency power supply, is incorporated. The advantage of this torch is that an 80 to 85% level of energy coupling efficiency is achieved and is sustained over a long-term service period. A water-cooled stainless steel probe penetrates the torch head to inject the powder axially for processing in the plasma.

**Figure 1:**
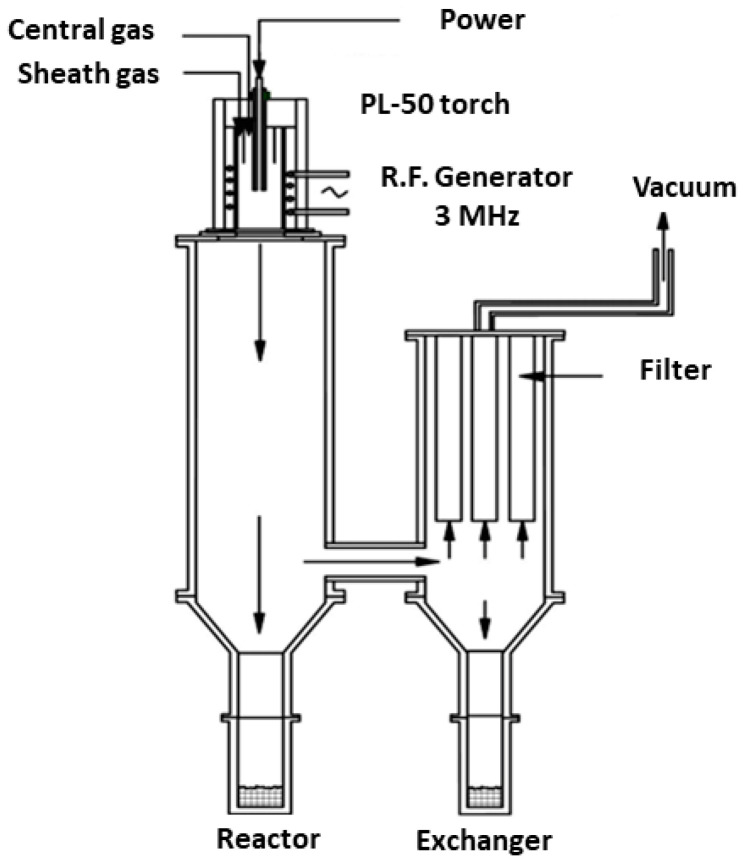
Schematic of the powder synthesis reactor.

The powder synthesis reactor, as shown in Figure 1, is designed to permit the efficient collection of the in-flight melted, and then resolidified powder that is mainly deposited on the larger chamber wall. A chamber side port, located at the base of the larger cylinder, is connected to the smaller cylinder where the secondary powder collection takes place, and where the ultra-fine powder is retained on the metal filter placed at the exit of the smaller cylinder chamber. Ultra-Large particles, which do not tend to deposit on the walls of the chamber, fall down into cylindrical containers attached to the lower part of the two main cylinders. These recipients are all intended to facilitate powder product collection. In this research, the powders accumulated on the reactor/chamber wall were collected for the studies.

The mechanically agglomerated Al-Mo and Al-Ni powders were fed to the reactor by means of a dry-injection circuit equipped with a gas flow disperser that was used to discourage the agglomeration of the particles in the feeding lines. The feed rate of agglomerated powder to the plasma reactor varied over the range of 6 to 13 g/min, depending on the used plasma conditions. Since the powder feeder interface does not provide much information, it was difficult to know the exact feed rate of the powder delivered to the plasma torch. The best way to acquire this information was to calculate it by knowing the amount of powder fed to the reactor and the elapsed feeding time. Before each plasma spray run, the spray chamber was evacuated to <50 torr pressure, neutral gas was then introduced to the torch, and the plasma was initiated. Following the stabilization of the plasma jet, the powder was injected via the probe. The different applied plasma parameters such as pressure, power, etc., are mentioned in the results and discussion part of the manuscript. The injected and plasma-processed powders were subsequently collected from the reactor wall for further characterization.

### 2.3. Separation of the Obtained Powder Particles

Based on the results, the powders produced by the plasma deposition consisted of a major portion with spherical large particles that were surrounded by porous particles. The surrounding porous particles are in fact agglomerates of nano-sized particles. Therefore, a separation operation was necessary to achieve adequate material for the characterization procedures. The separation was performed by washing the powder with methanol in an ultrasonic bath. The nano-sized particles stayed in suspension in methanol while the larger spherical particles were precipitated. In this process, the ultrasound apparatus was immersed in the suspension for 10 min to ensure sufficient time for particle separation. Following this procedure, the larger particles were found at the base of the beaker while the nanoparticles remained in methanol suspension.

### 2.4. Characterization of the Obtained Powder Particles

The microstructural images of processed powders (based on the emissions received from the BSE) were obtained using the mentioned SEM machine equipped with an EDX system.

To identify the crystalline phases resulting from possible reactions that occurred during the plasma process between the two elements existing in each of the Al-Mo and Al-Ni powder systems, phase analysis of the product powders was performed by XRD technique on a computer-controlled Philips X’pert Pro unit (Amsterdam, The Netherlands). The identification of the present crystalline compounds, even in quite complex samples, was made by the JADE 7.0, an application for the characterization of products. This application can quickly and accurately lead to the identification of the phases producing the generated patterns. In addition, the EDX system of the SEM machine was also employed to obtain information on elemental distribution and the elemental ratio at different spots and regions of the powder particles. This technique also leads to the identification of different phases existing in powder particles.

## 3. Results and Discussion

### 3.1. Characterization of the Initial Powders

**Figure 2:**
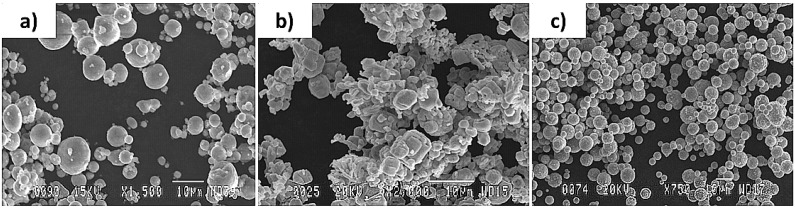
Image showing the microstructures of (**a**) Al powder particles, (**b**) Mo powder particles, and (**c**) Ni powder particles.

The general particle morphology of the Al powder was, as presented in Figure 2a, spherical. The powder particle size was in the range of 4.5–7 μm. The particle morphology of Mo powder was irregular, as illustrated in Figure 2b, and the average particle size was 1–5 μm. However, some particle agglomeration was also present in the delivered product. The morphology of the Ni powder was spherical, as shown in Figure 2c and its average particle size was about 6.6 μm. Similar to the Mo powder, some agglomeration, being formed through the chain formation that possibly took place due to the magnetic properties of Ni, was also seen to be present in this powder.

Table 1 reports the amounts of O and C impurities existing in the used powders. This information might be of use in analyzing the reasons for the formation of different phases in the obtained final powders.

### 3.2. Mechanical Agglomeration of the Powders

The objective of this section was to understand the optimum required time to synthesize the Al-Mo and Al-Ni composite powders having the discussed required properties.

**Figure 3:**
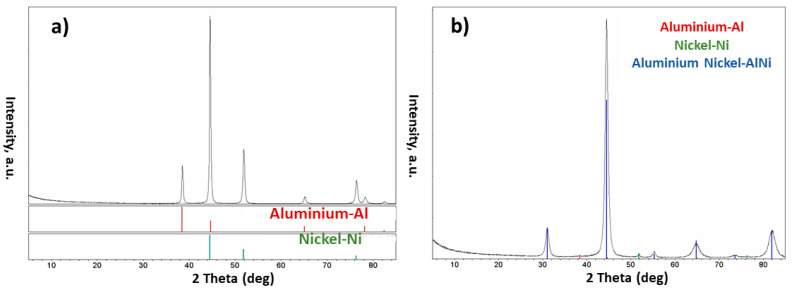
XRD results obtained from the Al-Ni powder system after (**a**) 6 min, 1 h, and 3 h, and (**b**) 10 h of the milling process.

**Figure 4:**
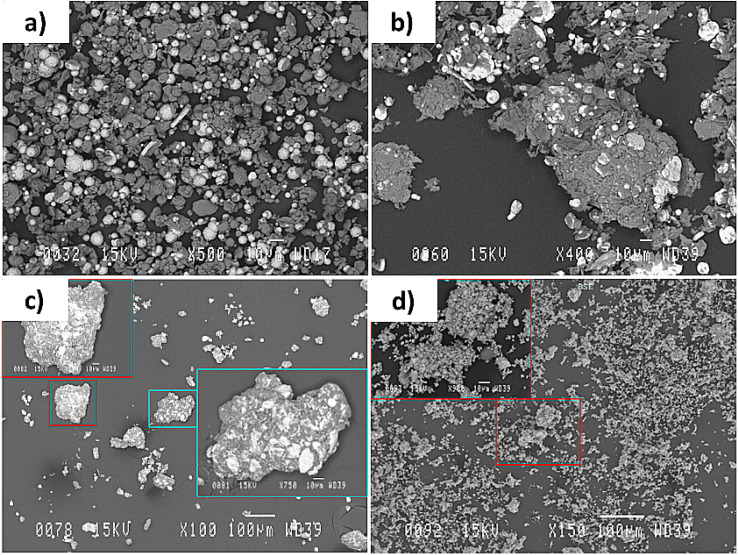
SEM images of the Al-Ni powder system being milled for (**a**) 6 min, (**b**) 1 h, (**c**) 3 h, and (**d**) 10 h. Note that the particles existing in the larger rectangles show the enlarged microstructure of the ones existing in the smaller rectangles.

The first tests using the ball milling apparatus were made on the Al-Ni system. After applying 6 min or 1 h of milling, the only peaks detected in the XRD patterns were related to the two pure elements, namely Al and Ni (Figure 3a). This means that no alloy was formed during these milling processes, which is what we desire. The differences related to the milling time come from the SEM analysis showing that the effect of milling time on the grains was appreciably different (Figure 4). Milling for 6 min was not sufficient; this is because almost no effect was observed on the powder particles and the Al and Ni particles were not agglomerated (Figure 4a). For 1 h of the milling process, the obtained grains were, in general, coarse. However, the homogeneity of elemental components inside the grains was not sufficient (Figure 4b). Nevertheless, since the Al particles adopted a flake shape, it can be concluded that 1 h of the milling process was successful to affect the Al particles. The Al and Ni particles tend to become deformed plastically during the shocks: the powders are either trapped between the two balls or are trapped between a ball and the jar wall. This phenomenon hardens the particles and then involves their rupture. It leads to the creation of new extremely reactive surfaces and welding occurs between these surfaces during collisions. The repeated shocks result in particles being rolled (flattened), fractured, and welded in a repeated way. Once the balance is reached between the coalescence and the fragmentation of the particles, there is the stabilization of crystallite sizes [22].

In the case of 3 h of the milling process, the results were reproducible, and only peaks related to pure elements were observed in the XRD patterns (Figure 3a). On the contrary, once 5 h of the milling time was conducted, the results were not conclusive; in this sense that alloy formation was observed in some cases and not in others. As a result, 5 h of the milling process seems to be the time limit for the alloy formation. The morphology of the powder particles after 3 h of milling was observed under the SEM. During this milling process, there was the appearance of a lamellate structure made up of Al and Ni and the homogeneity between the Al and Ni elements inside the grains was relatively good (Figure 4c). Note that the BSE image color is linked to the density of compounds. Thus, bright areas indicate the presence of the heavier component that is Ni, and the darker ones to the presence of Al. In the case of 10 h of milling, the results were very reproducible. The milling process provided sufficient energy to the system to react and form the alloy. XRD results showed the presence of the AlNi phase (Figure 3b). In the same way, the SEM pictures revealed a change in the morphology of particles as compared to those being milled for 3 h (Figure 4c,d). The composite microstructure consisted of fine particles with a highly porous structure and an irregular granular morphology (Figure 4d). The 3 h of milling was preferred over the 1 h option. This is because of the size and form of the grains after the milling process and the homogeneity of Al and Ni elements existing inside each grain of this composite powder system.

Based on the results obtained from the SigmaScan Pro software, the mean size of the large particles in the powders milled for 3 h was about 70 µm, which is above the required size (about 30 µm), and the mean size of the smaller particles was about 8 µm.

**Figure 5:**
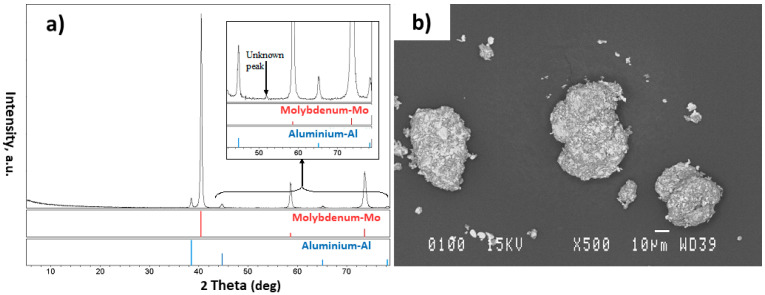
Image showing the (**a**) XRD pattern and (**b**) the SEM obtained microstructure of Al-Mo powder system milled for 3 h.

Based on the obtained results, 3 h of milling time was considered as the optimum time to agglomerate the non-alloyed Al-Ni powder system and was also used to mill and agglomerate the Al-Mo powder particles. Results obtained from the XRD and SEM tests were promising for this powder system (Figure 5). Excluding the one small unknown peak, only the diffraction lines for the pure element phases were present in the XRD patterns (Figure 5a). This means that the elemental powders did not react together, and no alloy was formed between the Al and Mo elements. Analyses carried out by the SigmaScan Pro software showed that the powder particles were greater than about 30 µm and the homogeneity between the Al and Mo elements was satisfactory (Figure 5b). Concerning the small particles, which can also be seen in Figure 5b, the inside distribution of Al and Mo appeared also sufficient and these particles are also expected to consequently result in alloy formation during the plasma treatment.

### 3.3. Plasma Deposition Results

Table 2 shows the parameters applied during the plasma treatment of the Al-Ni agglomerated powders. Note that these parameters were chosen based on previous experiments.

**Figure 6:**
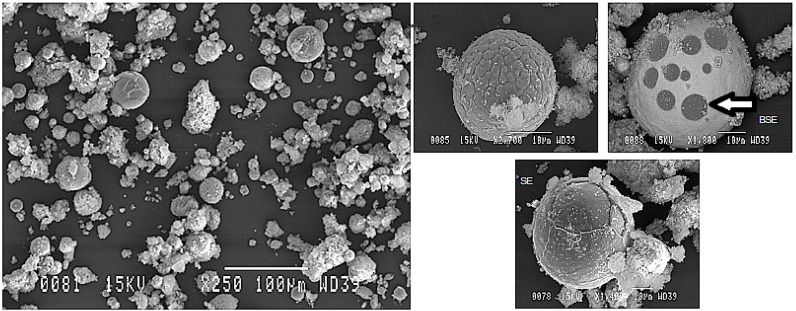
SEM image of the Al-Ni powder particles obtained from the reactor wall. The powder was processed using the parameters mentioned in Table 2. The arrow indicates an example of an Al spot trying to diffuse to the Ni.

Figure 6 shows the powder particles collected from the reactor. The powder appeared to consist of two different kinds of particles. That is, larger spherical particles, their sizes ranging from 2 µm to 40 µm, were coated in a composite microstructure made up of very fine particles. These fine particles are estimated to be condensed from the vapor phase. In order to determine the real amount of large particles versus fine particles deposited on the reactor wall, it was decided to separate the particles according to size using the separation procedure. The results appear to be good since the large particles represented about 75% of the powder mass before commencing the separation procedure. Some of the large spherical particles, especially those with a diameter greater than 20 µm, showed different aspects as compared to the other large ones. In essence, some spots were observed on the surface of these particles (as indicated by the arrow in Figure 6). Considering that these images were obtained in BSE mode, these spots were estimated to be the initially used Al particles that were trying to diffuse into the molten Ni particles during the plasma treatment process.

**Figure 7:**
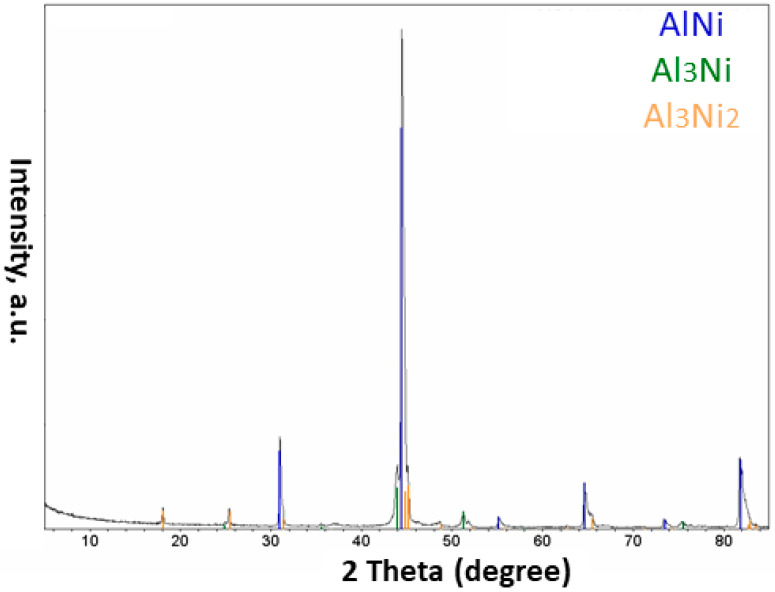
XRD pattern of the powder obtained after applying the plasma treatment (Table 2) to the Al-Ni powder system.

Based on the results obtained from the XRD tests done on the processed powder (Figure 7), the Al and Ni elements existing in the initial powder agglomerates had reacted together resulting in the formation of different Al_x_Ni_y_ intermetallic phases in the plasma-treated particles. These phases included AlNi, AlNi_3_, and Al_3_Ni_2_.

**Figure 8:**
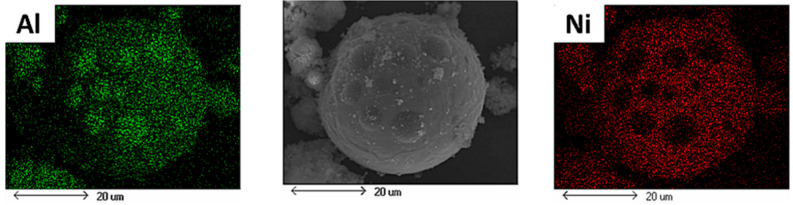
EDX analysis of the powder particles obtained after applying the plasma treatment to the Al-Ni powder system.

EDX analysis was performed on such particles to evaluate the BSE image observations and the results of XRD tests. As seen in Figure 8, this analysis clearly indicated that the fine and large particles were consisting of both Al and Ni. This is another piece of evidence confirming the success of the plasma treatment process to mix the initially used elements together and form new phases. In addition, as the intensity of Ni (red in Figure 8) was very low in the EDX tests of the surface spots, the results show that the spots on the surface of large particles were pure Al.

Identical parameters, as those used for the Al-Ni powder system (Table 2), were applied to treat the Al-Mo agglomerated powders. However, it was concluded that 20 kW of power is too much for the Al-Mo powder system leading to extensive evaporation during the process. Table 3 shows the new set of parameters applied during the plasma treatment of the Al-Mo agglomerated powders. Note that the decreased power was the only difference between this set of parameters with that used for the Al-Ni powder system (Table 2).

**Figure 9:**
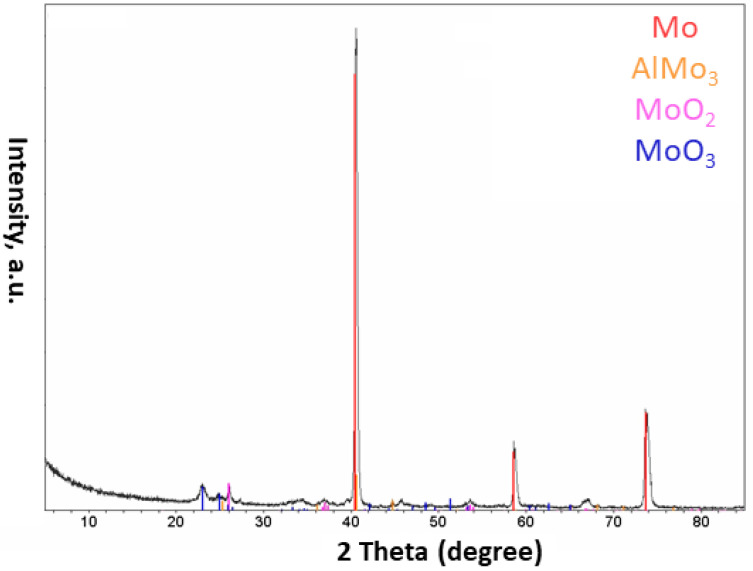
XRD pattern of the powder obtained after applying the plasma treatment with parameters mentioned in Table 3 to the Al-Mo powder system.

The obtained results were interesting. The first observation was related to the color of the as-collected powder. The plasma-treated powder was blue and no longer dark grey; it was a completely different color from that of the initially used powder. According to the color characteristics of the MoO_2_, it was thought that the product powder included this type of oxide inside. XRD experiments (Figure 9) confirmed this understanding. Based on the obtained results, the synthesized power consisted of different phases including Mo, AlMo_3_, MoO_2_, and MoO_3_.

**Figure 10:**
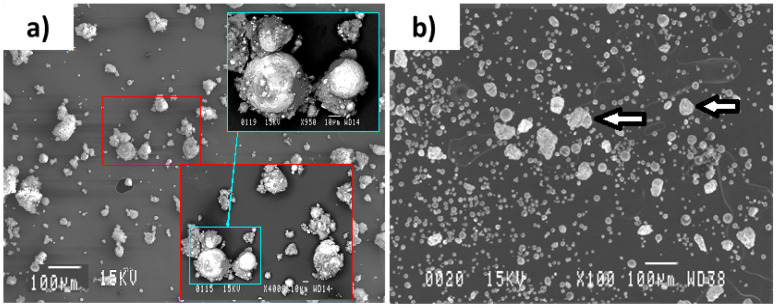
SEM image showing (**a**) the Al-Mo powder particles obtained from the reactor wall and (**b**) the larger particles obtained after doing the separation process. The powder was processed by the parameters mentioned in Table 3. Note that the particles existing in the larger rectangles show the enlarged microstructure of the ones existing in the smaller rectangles. The arrows show the particles appearing similar to their initial agglomerated non-treated condition.

Similar to the SEM image of the Al-Ni plasma processed powder (Figure 6), the SEM image of the Al-Mo version (Figure 10a) shows the existence of several large particles being hidden or covered within a cloud of very fine particles. Additionally, similar to those of Al-Ni ones, Al spots were seen on the surface of most of the large particles. This indicates that the process of Al diffusion into the molten Mo particles was not completed.

To have a better understanding of the types of particles existing in the powder and the reason for their color being blue, the powder was separated into various size ranges. After performing the separation treatment, the first observation made was that the larger particles were grey in color. The collected finer particles recovered from the separation treatment were blue in color, suggesting that the discussed oxide phase was amongst these particles. Under the plasma suspension feeding condition, most of the input particles were fully melted while some were completely evaporated. These particles, being condensed from the vapor phase, consisted of fine particles and had highly porous structures with mainly irregular morphology. The larger particles (Figure 10b), as separated by the discussed procedure, could be further separated into two types of particles: Particles of 5–40 µm in size and being spherical, and the ones which appeared similar to their initial agglomerated non-treated condition (as indicated by the arrows in Figure 10b). This type of particle is generated when the melting conditions are so inadequate that the initially used particles do not get fully melted and they consequently stay non-reacted.

Nevertheless, because of the oxide production, a new plasma deposition process was undertaken using a mixture of He and Ar gases, instead of Ar being solely used as the gas. Parameters used for this set of experiments are presented in Table 4.

**Figure 11:**
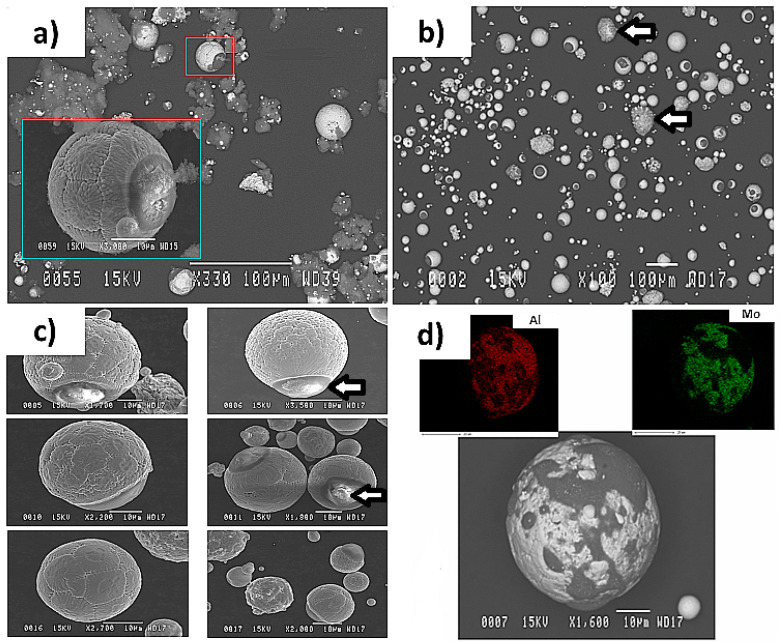
SEM images showing (**a**) the Al-Mo powder particles obtained from the reactor wall, (**b**) the larger particles obtained after doing the separation process. The arrows show the particles appearing similar to their initial agglomerated non-treated condition. (**c**) Larger particles with the flower-type morphology. The arrow indicates examples of an Al spot trying to diffuse to the Mo. (**d**) The EDX result of the reacted powder particles. The powder was processed using the parameters mentioned in Table 4. Note that the particles existing in the larger rectangles show the enlarged microstructure of the ones existing in the smaller rectangles.

Following the plasma deposition, the collected powder was dark grey in color, as it was with the initially used agglomerated powder or the Al-Ni plasma treated collected powder. There was also no more oxide phase detected in the XRD patterns and just the peaks related to Al, Mo, and AlMo_3_ were detected. These show that the new set of plasma parameters was successful to prevent the oxide formation in the treated powder, but not successful to lead in the full reaction between elements. As similar to the previous sets of tests, the SEM analysis images show the existence of several large particles being hidden within a cloud of very fine particles (Figure 11a). The results obtained from the separation procedure show that, the same as that of Al-Ni plasma treated powder, the large particles represented about 75% of the powder mass before commencing the separation procedure (Figure 11b). The morphology and characteristics of these separated larger particles were similar to those of the Al-Mo ones processed under the parameters mentioned in Table 3 (Figure 10b).

These separated larger particles contained non-reacted particles, which were identical to the initially used agglomerated particles (as indicated by the arrows in Figure 11b), and several particles with flower-type morphology (Figure 11c). These particles are generated when the melting conditions are so inadequate that particles retain their polygonal shapes, rather than being fully melted and having completely spherical and smooth surfaced forms. The enlarged picture of this type of particle reveals the limits of immiscibility existing between Al and Mo, as the Al is seen as spots trying to diffuse inside Mo (as indicated by the arrows in Figure 11c). The particles containing reacted parts were identified within the largest particles and their sizes could reach 40 µm in diameter (Figure 11d). As the results of EDX analysis showed, a portion of these kinds of particles contained both Al and Mo components in their composition (Figure 11d).

Considering the above results, it appears that there was no significant interaction between Al and Mo during the designed plasma processes. It also appears that the level of interaction between Al and Mo was less than that of Al and Ni. This is because the elemental Al and Mo phases were detected and largely present in the obtained particles processed by the discussed set of parameters (Table 3 and Table 4). Al seems to have become evaporated during the plasma process and there was no significant interaction between the Al vapor and the molten Mo particles. To generate more Al_x_Mo_y_ type phases, it is recommended that future works should focus on using initial particles with Al/Mo mass ratios of over 1.

## 4. Conclusions

In this research, plasma spraying was conducted on Al-Ni and Al-Mo non-alloyed agglomerated powder systems. This was done to synthesize different Al alloys and intermetallics by implementing the plasma system on these two different powder systems. The initially used agglomerated powder systems were obtained from blends of elemental Al and Ni or Al and Mo using the milling process. The mass ratio existing between each of these elements was 1.

The following results were obtained:

1. The variation of 3 h of milling time was found to be the optimum time to agglomerate and form non-alloyed Al-Ni and Al-Mo powder systems having a homogeneous distribution of elements in their particles.
2. The injection of each of these composite powders into the plasma system resulted in the formation of different phases in the obtained particles. Different phases such as Al, AlNi_3_, Al_3_Ni_2_, and AlNi were detected in the particles of the Al-Ni powder system, and Al, Mo, AlMo_3_, MoO_3_, and MoO_2_ in the Al-Mo powder system
3. The powders collected from the reactor wall consisted of large particles being covered within a cloud of very fine particles. The fine particles are thought to be condensed from the vapor phase and the reacted large particles were mostly seen to have Al spots on their surfaces. This shows the diffusion of molten Al into the molten Mo or Ni and was interpreted as a sign of immiscibility of Al into these molten elements
4. Particles appearing similar to their initial agglomerated non-treated condition, being absent in the Al-Ni particles, were also seen amongst the large particles of the Al-Mo powder system. This indicates that the level of reaction between Al and Mo was less than that of Al and Ni during the plasma processes.
5. It was noted that using a mixture of He and Ar gases, instead of solely using the Ar gas, could prevent the formation of oxides in the Al-Mo plasma treated powder system.

## Figures and Tables

**Table 1:** Amounts of O and C impurities existing in the used powders.

Powder	O (wt.%)	C (wt.%)
Al	0.05	0.06
Mo	0.003	0.002
Ni	0.04	0.05

**Table 2:** Table showing the parameters applied to plasma treat the Al-Ni agglomerated powders. Note that the gas used in these experiments was Ar.

Sheath Gas (L/min)	Central Gas (L/min)	Carrier Gas (L/min)	Pressure (Torr)	Power (kW)
80	25	6	300	20

**Table 3:** Table showing the parameters applied to plasma treat the Al-Mo agglomerated powders. Note that the gas used in these experiments was Ar.

Sheath Gas (L/min)	Central Gas (L/min)	Carrier Gas (L/min)	Pressure (Torr)	Power (kW)
80	25	6	300	10

**Table 4:** Table showing the parameters applied to plasma treat the Al-Mo agglomerated powders. Note that the gas used in these experiments was a mixture of Ar and He.

Sheath Gas (L/min)	Central Gas (L/min)	Carrier Gas (L/min)	Pressure (Torr)	Power (kW)
Ar: 15-He: 100	Ar: 25	He: 3	300	10

## Data Availability

The datasets generated during and/or analysed during the current study are available from the corresponding author on reasonable request.
